# Hydrogen sulfide facilitates reprogramming and trans-differentiation in 3D dermal fibroblast

**DOI:** 10.1371/journal.pone.0241685

**Published:** 2020-11-12

**Authors:** Elena A. Ostrakhovitch, Shin Akakura, Siamak Tabibzadeh

**Affiliations:** Frontiers in Bioscience Research Institute in Aging and Cancer, Irvine, CA, United States of America; Università degli Studi della Campania, ITALY

## Abstract

The efficiency of cell reprogramming in two-dimensional (2D) cultures is limited. Given that cellular stemness is intimately related to microenvironmental changes, 3D cell cultures have the potential of overcoming this limited capacity by allowing cells to self-organize by aggregation. In 3D space, cells interact more efficiently, modify their cellular topology, gene expression, signaling, and metabolism. It is yet not clear as how 3D culture environments modify the reprogramming potential of fibroblasts. We demonstrate that 3D spheroids from dermal fibroblasts formed under ultra-low attachment conditions showed increased lactate production. This is a requisite for cell reprogramming, increase their expression of pluripotency genes, such as *OCT4*, *NANOG* and *SOX2*, and display upregulated cystathionine-β-synthase (*CBS*) and hydrogen sulfide (H_2_S) production. Knockdown of *CBS* by RNAi suppresses lactic acid and H_2_S production and concomitantly decreases the expression of *OCT4* and *NANOG*. On the contrary, H_2_S donors, NaHS and garlic-derived diallyl trisulfide (DATS), promote the expression of *OCT4*, and support osteogenic trans-differentiation of fibroblasts. These results demonstrate that *CBS* mediated release of H_2_S regulates the reprogramming of dermal fibroblasts grown in 3D cultures and supports their trans-differentiation.

## Introduction

The appeal of regenerative medicine lies in the use of stem cells to replace damaged tissues. The patient-specific pluripotent stem cells are generated by de-differentiation of adult somatic cells. The reprogramming is achieved by the introduction of a combination of Octamer Binding Transcription Factor 4 (*OCT4* also called *POU5F1*), *KLF4*, SRY-Box transcription factor 2 (*SOX2*) and *cMYC* or *OCT4* (*POU5F1*), *KLF4*, *SOX2* and *NANOG* to dermal fibroblasts [1–3]. iPSCs have also been generated by the use of small-molecule drugs [4]. Artificially induced pluripotent cells (iPSCs) differentiate into cells of all three germ layers. In view of such vast differentiation potential, iPSCs have gained many clinical applications in regenerative medicine. However, iPSCs therapy faces significant difficulties such as low efficiency and safety issues at the risk of tumorigenesis following implantation. These issues limit the therapeutic values of iPSC.

Reprogramming of somatic cells is a multi-step process characterized by transcriptome and proteome resetting [5]. The initial phase of reprogramming is characterized by cytoskeletal changes [6]. In 3D conditions, extracellular physical forces provide a crucial set of signals that control cell structure and functions. These forces facilitate cell reprogramming and increase the efficiency of trans-differentiation. Transient acquisition of pluripotency (without progressing through iPSC intermediates) reprograms fibroblasts to a different somatic cell type that facilitates the lineage trans-differentiation [7]. In addition, studies using organoid culture showed that tissue-specific stem cells in 3D culture exhibit better tissue-specific function and more easily reproduce the *in vivo* tissue development and regenerative processes [8].

The 3D culture enhances the reprogramming of human mesenchymal stem/stromal cells (hMSCs) into iPSCs due to upregulation of the pluripotency factors such as *OCT4*, *SOX2*, and *NANOG*, however, as compared with embryonic stem cells, the expression levels of these genes are relatively low [9, 10]. MSCs show a high differentiation potential when cultured in 3D as compared to 2D condition. It was suggested that the MSCs cultured under 3D conditions, rather than reaching a pluripotent state, perhaps transit through a developmental stage that is equivalent to an early mesoderm. Similar to bone marrow MSCs, standard 2D culture of human dental pulp stem cells (DPSCs) affects stemness markers expression and proliferation rate [11]. 3D culture conditions were favorable to preserving DPSCs biological properties [12]. It was demonstrated that similar to cells that are reprogrammed towards pluripotency during 3D de-differentiation the cells switched from oxidative phosphorylation (OXPHOS) to an anaerobic-type metabolism [13]. Human dermal fibroblasts (HDFs) which share *in vitro* MSCs morphology, express MSCs markers and show the potential to differentiate along the three main mesenchymal-derived tissues are easier to obtain [14, 15]. In addition, HDFs have a higher proliferation rate. For these reasons, HDFs are a more practical alternative to MSCs for creation of tailor-made tissues. The efficacy of dermal fibroblasts to differentiate to osteoblasts is low, as compared to MSCs.

Transcriptome analysis showed that dermal fibroblasts which were grown in 3D as spheroids express pluripotency genes such as *OCT4*, *SOX2*, *NANOG*, and *Lin28* and resemble somatic cells that are reprogramed into stem cells [16]. Here, we show that human dermal fibroblasts cultured in 3D as spheroids express pluripotency genes, de-differentiate and trans-differentiate to osteoblasts by a program that requires cystathionine-β-synthase (*CBS*) mediated release of hydrogen sulfide (H_2_S).

## Materials and methods

HDFs were obtained from ATCC (Manassas, VA). Chemicals were from Sigma-Aldrich Company (St Louis, MO) or Fisher Scientific (Pittsburgh, PA). siRNAs to *CBS* were purchased from Santa Cruz Biotechnology (Paso Robles, CA). Polystyrene suspension culture dishes were obtained from Corning (Santa Barbara, CA). Primers were purchased from IDT (San Diego, CA). Sequences of primers used for gene amplifications are shown in Table 1.

**Table 1:** Real-time qPCR primers.

Gene	Forward primer (5’- 3’)	Tm	Reverse primer (5–3’)	Tm
β-ACTIN	TTG CCG ACA GGA TGC AGA AGG A	61	AGG TGG ACA GCG AGG CCA GGA T	65
c-MYC	AAA CAC AAA CTT GAA CAG CTA C	51.8	ATT TGA GGC AGT TTA CAT TAT GG	51.4
OCT4A	CTC CTG GAG GGC CAG GAA TC	59.8	CCA CAT CGG CCT GTG TAT AT	54.4
KLF4	ACC AGG CAC TAC CGT AAA CAC A	58.5	GGT CCG ACC TGG AAA ATG CT	57.7
SOX2	CCC AGC AGA CTTC ACA TGT	55	CCT CCC ATT TCC CTC GTTTT	55.1
NANOG	TGA ACC TCA GCT ACA AAC AG	52.3	TGG TGG TAG GAA GAG TAA AG	51
TET1	CAG AAC CTA AAC CAC CCG TG	64.2	TGC TTC GTA GCG CCA TTG TAA	67.5
CBS	TCA AGA GCA ACG ATG AGG AG	54.4	ATG TAG TTC CGC ACT GAG TC	54.7
CSE	AGA AGG TGA TTG ACA TTG AAG G	61.9	CAA TAG GAG ATG GAA CTG CTC	60.3
MPST	CGC CGT GTC ACT GCT TGA T	58.3	CAC CTG GAA GCG CCG GGA TT	62.9
DNMT1	CCT AGC CCC AGG ATT ACA AGG	61.4	ACT CATC CGA TTT GGC TCT TTC	60.4
RUNX2	ATG CGC CCT AAA TCA CTG AG	55.1	GTC TTC ACA AAT CCT CCC CA	54.5
OCN	GTG CAG AGT CCA GCA AAG GT	57.8	AGC AGA GCG ACA CCC TAG AC	58.8
ALP	AAC GTG GCC AAG AAC ATC ATC A	57.2	TGT CCA TCT CCA GCC GTG TC	59.4

### Cell culture and viability assay

HDFs were cultured in Dulbecco’s Modified Eagle Medium (DMEM) supplemented with 10% fetal bovine serum (FBS) and 1% penicillin and streptomycin (P/S) at 37°C in and atmosphere with 5% CO_2_. HDFs were trypsinized and passaged at a ratio of 1:3.

### 3D culture conditions

Prior to confluency, HDFs were trypsinized. Cells were resuspended in the αMEM supplemented with 20% Knockout Serum Replacement (ThermoFisher Scientific), 5 ng/ml basic FGF (GoldBio, St Louis MO), 10 ng/ml EGF and 0.1 mM NEAA culture media and seeded in low attachment plates to promote self-assembly and formation of spheroids. Cells were incubated at 37°C with 5% CO_2_ for 3–6 days.

### Disaggregation of 3D spheroids

Spheroids were removed from the culture plates and settled by centrifugation at 180 xg for five minutes at 4°C. Spheroids were washed in 5ml 1x PBS, centrifuged and then then were resuspended in 0.25 mg/ml collagenase I and 1U/ml DNase and incubated at 37°C for five minutes. Spheroids were disaggregated by gentle pipetting for no longer than 15 minutes. Single cells were resuspended in serum-¬containing cell culture medium.

### Transfection and RNA interference

For transfection, 2-5x10^5^ cells were cultured in DMEM medium without serum or antibiotics. Then, K2 transfection reagent (Biontex, Germany) complexed with targeting (siRNA) and non-targeting scrambled (Scr) control RNAs (Cruz Biotechnology, Paso Robles, CA) were added to cell cultures. Each oligonucleotide was dissolved in 100 μM Duplex Buffer (100 mM Potassium Acetate, 30 mM HEPES, pH 7.5) and mixed in equal molar amounts, at a final concentration of 10 μM per oligonucleotide. Oligonucleotides were annealed at 94°C for 2 minutes and then cooled to room temperature for 2 hours. After 48 hours of transfection, the medium was replaced with αMEM medium supplemented with knockout serum replacement medium and cells were treated for 3 additional days prior to being harvested for analysis.

### RNA extraction, cDNA synthesis and quantitative real-time PCR

Total RNA was extracted from cultured cells with TRI reagent (Sigma-Aldrich, St Louis, MO) following the manufacture’s instruction. Total RNA (1 μg) was reverse transcribed in a final volume of 20 μL using Thermo Scientific (Gaithersburg, MD) cDNA synthesis kit. 5 ng cDNA was used in 10 μL reactions for quantitative real time PCR (qPCR). qPCR reaction mixture consisted of 5 μl iTaq Universal SYBR Green supermix (Bio-Rad, Hercules, CA), 100 nM upstream primer, 100 nM downstream primer, 5 ng cDNA. qPCR was performed using LightCycler 96 system (Roche Diagnostics, Indianapolis, IN). The reaction conditions for qPCR were as follows: initial denaturation step at 95°C for 10 min, 40 cycles of 95°C for 15 sec, 60°C for 45 sec, followed by melting curve analysis. The gene expression by qPCR was analyzed by relative quantification using 2^−ΔΔCt^ method as described previously [17, 18]. Data were processed using Roche Real-Time PCR Analysis Software LightCycler 96 SW1.1 (Roche molecular diagnostics, Pleasanton, CA). Relative expression level for each gene was normalized to that of *β-ACTIN* as a housekeeping gene.

### Biochemical analysis

As we reported recently, H_2_S levels were quantified by using Free Radical Analyzer (TBR4100 and ISO-H_2_S-2, World Precision Instruments, Sarasota, FL) in accord with the manufacturer’s instruction [19]. Prior to the measurements, the sensor was polarized and calibrated by adding four aliquots of the Na_2_S stock solution at the final concentrations of 0.25, 0.5, 1 and 2 μM. Lactic acid was quantified by a spectrophotometric method using 1% p-phenylphenol as described previously [20].

### Immunostaining

HDFs were washed in PBS and then fixed using 4% paraformaldehyde for 20 minutes. HDFs were permeabilized with 0.2% Triton X-100 for 30 minutes and then stained with rhodamine-phalloidin at a dilution of 1:1000. HDFs were then washed with PBS and the cells were imaged using a light microscope (Olympus, Model IX50).

Fluorescence imaging of H_2_S in live cells was obtained with H_2_S fluorescent probe HSN2 (a kind gift of Professor Michael D. Pluth, University of Oregon, Department of Chemistry, Eugene, Oregon) by incubating cells in 5 μM HSN2 for 30 min [21].

Cell proliferation was assessed by immunofluorescence staining of 5-bromo-2′-deoxyuridine (BrdU) that was incorporated at 2 μg/ml into the DNA for 30 minutes. Following such exposure, cells were fixed in 4% paraformaldehyde in PBS for 30 min, followed by a treatment with 2N HCl for 30 min to separate DNA into single strands. Nonspecific staining was blocked by incubation of cells with 5% BSA in PBS for 1 h with gentle agitation. Then cells were incubated overnight with primary antibody to BrdU followed washing and then incubation with streptavidin-HRP conjugate. Following washing, staining was achieved by incubating cells in 0.05% 3,3’-diaminobenzidine (DAB) substrate in 0.01M PBS and with 0.015% H_2_O_2_.

### Osteogenic differentiation

For osteogenic differentiation, DMEM was supplemented with 10% FBS, 100 nM dexamethasone, 50 μM ascorbate-2-phosphate and 10 mM β-glycerophosphate (Sigma-Aldrich, St Louis, MO). Medium was changed every 4–5 days. Negative control cells received DMEM supplemented with 10% FBS. Cells were maintained at 37°C and 5% CO_2_. After 21 days, osteogenic differentiation was revealed by staining the intracellular calcium.

### Histochemical staining

Calcium deposits were stained with staining with Alizarin Red 2 and with alkaline phosphatase [22]. Cells were stained in 2% Alizarin Red staining at pH 4.2 and then Alizarin was extracted from cells and absorbance was measured at 405 nm using SpectaMAX Plus plate reader (Molecular Devices, San Jose, California).

For Alkaline phosphatase, cells were fixed in 4% paraformaldehyde for 5 minutes at room temperature, and then they were washed with PBS/0.1% Tween-20 for 5 minutes. Alkaline phosphatase was visualized in cells via histochemical detection with 4-nitroblue tetrazolium (NBT) as a substrate and 5-bromo-4-chloro-8-indolilphosphate (BCIP) as a coupler and then equilibration of the incubation medium with NTMT solution (100 mM Tris pH 9.5, 100 mM NaCl, 50 mM MgCl_2_, 0.1% Tween-20). Color reaction was developed by incubating cells in NBT/BCIP solution and after 40 minutes, reaction was stopped by washing cells in PBS. Cells were visualized with a digital camera attached to a phase-contrast microscope (Olympus, Model IX50).

### Statistical analysis

All experiments were performed, at least in triplicates, and were repeated at least three times. The results are presented as means ± SEM derived from at least three experiments. Statistical analysis was performed using two-group comparisons by means of Student's t-test. Error bars in figures represent the Standard Deviation of the mean. Statistical significance is shown as *: p≤0.05, **: p≤0.005, ***: p ≤0.0005.

## Results

### 3D culture conditions modify the metabolism and gene expression patterns in HDFs

**Fig 1:**
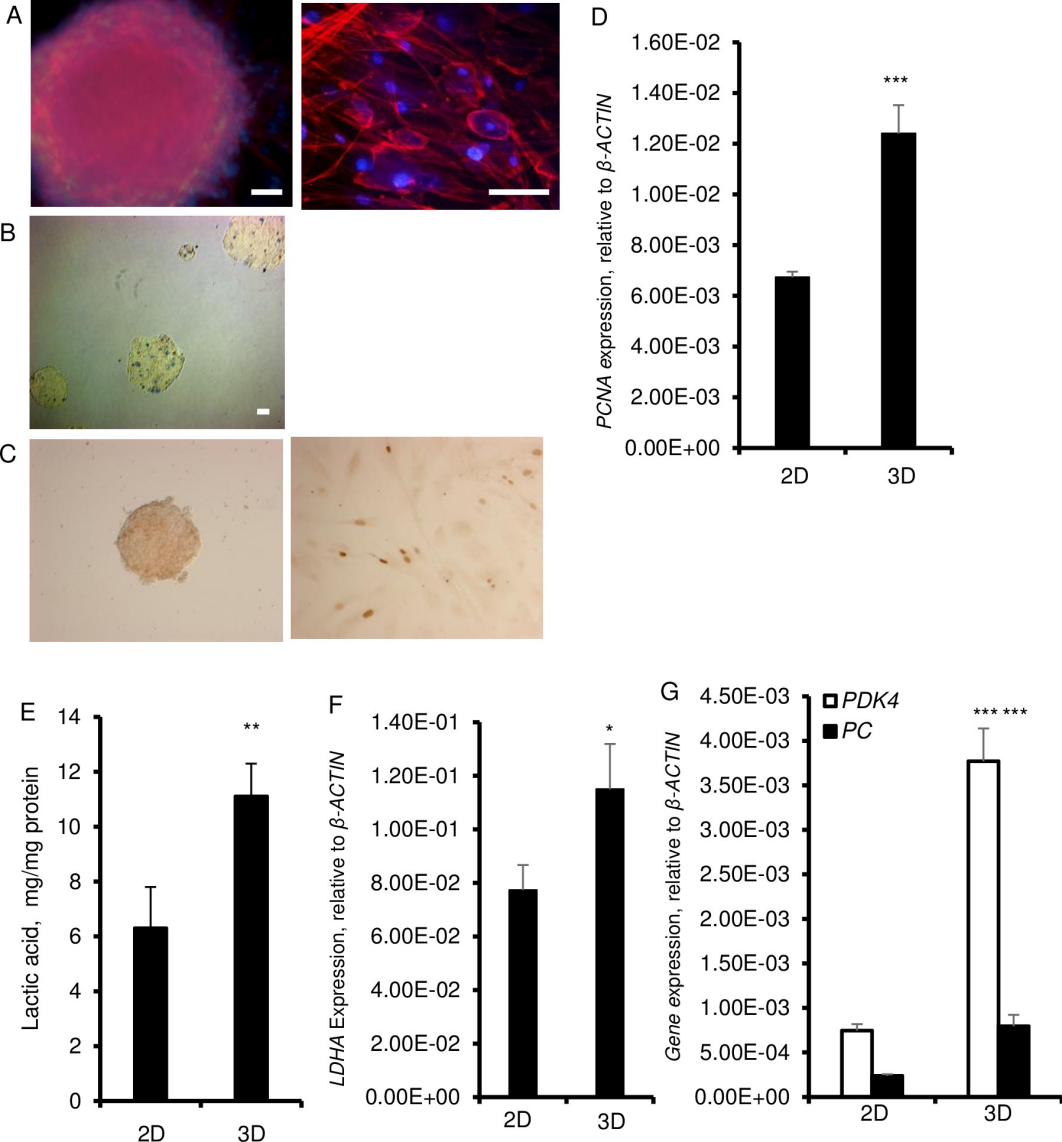
Cell morphology and metabolic features of fibroblasts grown in 3D. HDF were cultured in 2D and in 3D cultures on ultra-low attachment plates for 6 days in αMEM supplemented with 20% Knockout Serum Replacement, 5 ng/ml basic FGF, 10 ng/ml EGF and with 0.1 mM nonessential amino acids (NEAA). A. Staining of cytoskeletal F-actin with phalloidin (red fluorescence) in spheroids and monolayer cultures. Nuclei are stained with Hoechst (blue fluorescence). Spheroid was photographed at 20× magnification (left) and monolayer cultured cells were photographed at 40× magnification (right). Scale bars = 200 μm. B. Staining of spheroids with trypan blue. C. BrdU staining (brown) in HDF spheroids (left) and in 2D culture after 6 days. D. Expression of proliferating cell nuclear antigen (*PCNA*) at the mRNA level. *β-ACTIN* was used as a housekeeping control. E. Amount of lactic acid in supernatant over a 6-day culture period. F. Expression of *LDHA* mRNA. *β-ACTIN* was used as a as a housekeeping control. G. Expression of *PDK4* and *PC* mRNA. β-ACTIN was used as a housekeeping control. Data are shown as means ± SD from 3 independent experiments. Statistical significance is shown as * (p≤0.05), ** (p ≤0.005), and *** (p ≤0.0005) in 3D as compared to 2D cultured cells.

HDFs aggregated and formed spheroids within 24 hours when grown in suspension in polystyrene low attachment culture dishes in αMEM medium containing knockout serum replacement (Fig 1A–1C). The size of the spheroids increased over a 3-day period and remained the same for the rest of the culture period, up to 6 days. To adapt to this 3D condition, cells need to re-organize their cytoskeleton and establish tight junctions. In this work, we used phalloidin to examine the organization of actin filaments. Staining with fluorescently labeled phalloidin revealed spheroids of different sizes that developed a defined outer perimeter that showed compact bundles of microfilamentous F-actin along the cell periphery as stress fibers (Fig 1A). Within the spheroids, fibroblasts had a round morphology, a distinctly different cell shape different than their elongated morphology in 2D culture conditions. As established with Trypan blue, there were no necrotic cores and dead cells were randomly dispersed within the spheroids, particularly around day 6 (Fig 1B). Yet, on day 6, the cell proliferation was significantly higher and BrdU^+^ cells were scattered within spheroids and the number of proliferating cells was significantly higher in 3D than 2D cultures as evidenced by increased cell nuclear antigen (PCNA) (Fig 1C and 1D).

By virtue of compaction, we considered that nutrient depletion and hypoxia might force cells to develop glycolysis. To test this, we assessed glycolysis by virtue of measurement of lactate in the culture media. This analysis revealed that there were nearly two-fold higher amounts of lactate in 3D as compared to the 2D cultures (Fig 1E). Consistent with this, the expression of lactate dehydrogenase A (LDH-A) which catalyzes the conversion of pyruvate to lactate was also increased in 3D than 2D cultures (Fig 1F). Moreover, as compared to 2D cultures cells, spheroids exhibited high expression of pyruvate carboxylase (PC), which is necessary for glucose production, and pyruvate dehydrogenase kinase (PDK4), which affects the entrance of pyruvate to the TCA cycle (Fig 1G). Significantly elevated expression of PC and PDK4 in 3D cultures are consistent with the transition of metabolism to glycolysis.

### Reprogramming in HDFs grown in 3D culture is dependent on *CBS* expression and H_2_S production

**Fig 2:**
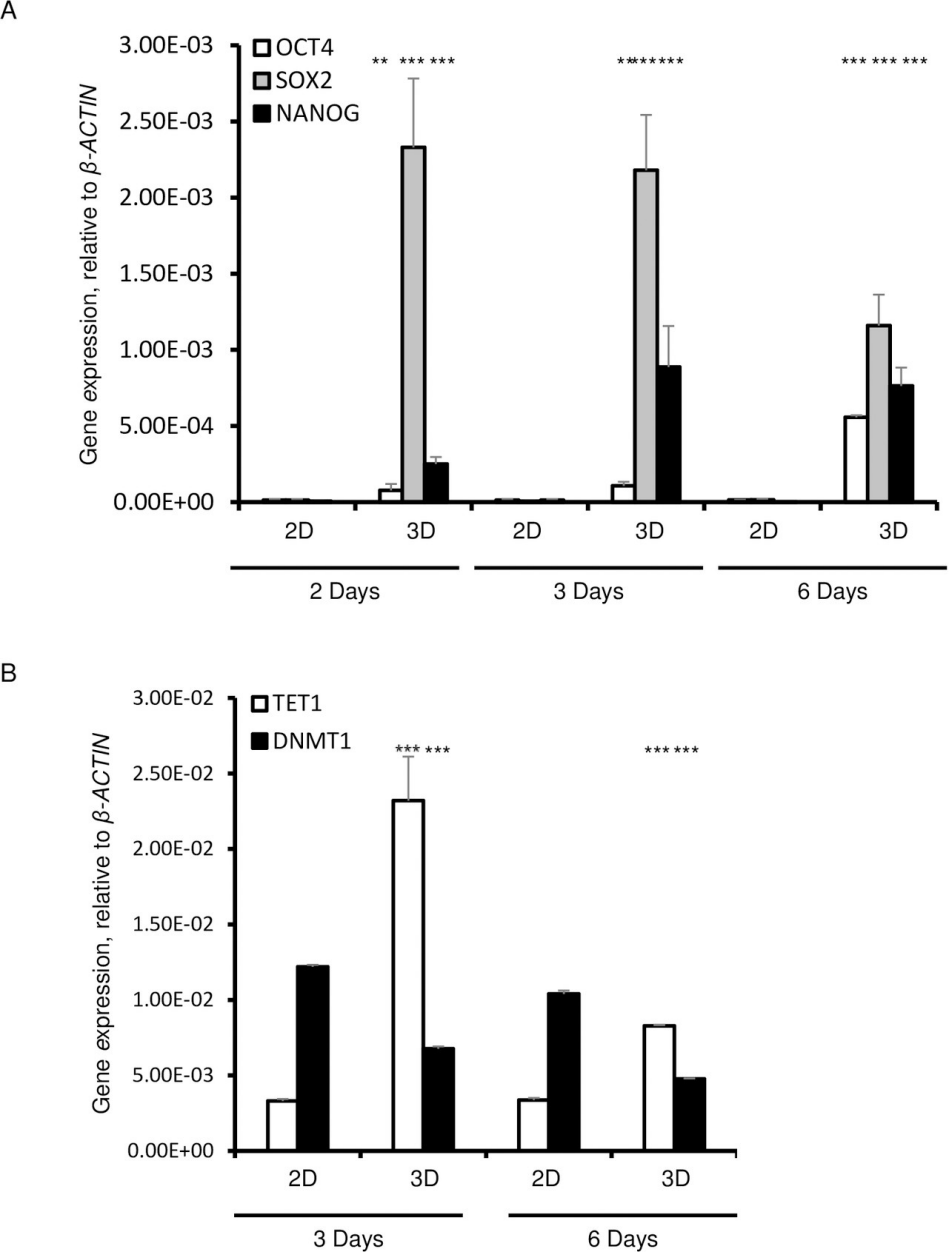
Expression of pluripotency markers, *OCT4*, *SOX2*, and *NANOG* in 3D spheroids cultured for 2, 3, and 6 days. A. qPCR of *OCT4*, *SOX2* and *NANOG*. B. q PCR analysis of *TET1* and *DNMT1*. Data are presented as the means ± SD from 3–6 independent experiments. *β-ACTIN* was used as a housekeeping control. Statistical significance is shown as * (p≤0.05), ** (p ≤0.005), and *** (p ≤0.0005) in 3D as compared to 2D cultured cells.

Since glycolytic metabolism is a requisite for the cells that reside in a pluripotent state, we analyzed the expression of pluripotency genes after 6 days of culture. The expression levels of *OCT4*, *NANOG*, and *SOX-2* were significantly upregulated in HDFs that were cultured in 3D as compared to those cultured in 2D conditions (Fig 2A). The expression of *NANOG* was significantly higher on day 2 and remained high on days 3 and 6 in 3D cultures (Fig 2B). Remarkably, the expression level of *OCT4* steadily increased from day 2 to day 6, while the expression of *SOX2* decreased from day 3 to day 6 (Fig 2B).

DNA methylation is a requisite step in the regulation of pluripotency genes [23]. For this reason, we examined the expression of Ten-Eleven-Translocation (*TET1*), which specifically catalyzes the conversion of the modified DNA base 5-methylcytosine (5-mC) to 5-hydroxymethylcytosine (5-hmC), and of DNA (cytosine-5)-methyltransferase 1 (*DNMT1*), an enzyme that catalyzes the transfer of methyl groups to specific CpG structures in DNA that is responsible for *de novo* DNA methylation [24, 25]. *TET1* expression was significantly increased, whereas the expression of DNA methyltransferases (DNMT1) was significantly decreased in 3D as compared with the 2D cultures that showed decreased expression of *TET1* and a significantly higher expression of *DNMT1* (Fig 2D).

**Fig 3:**
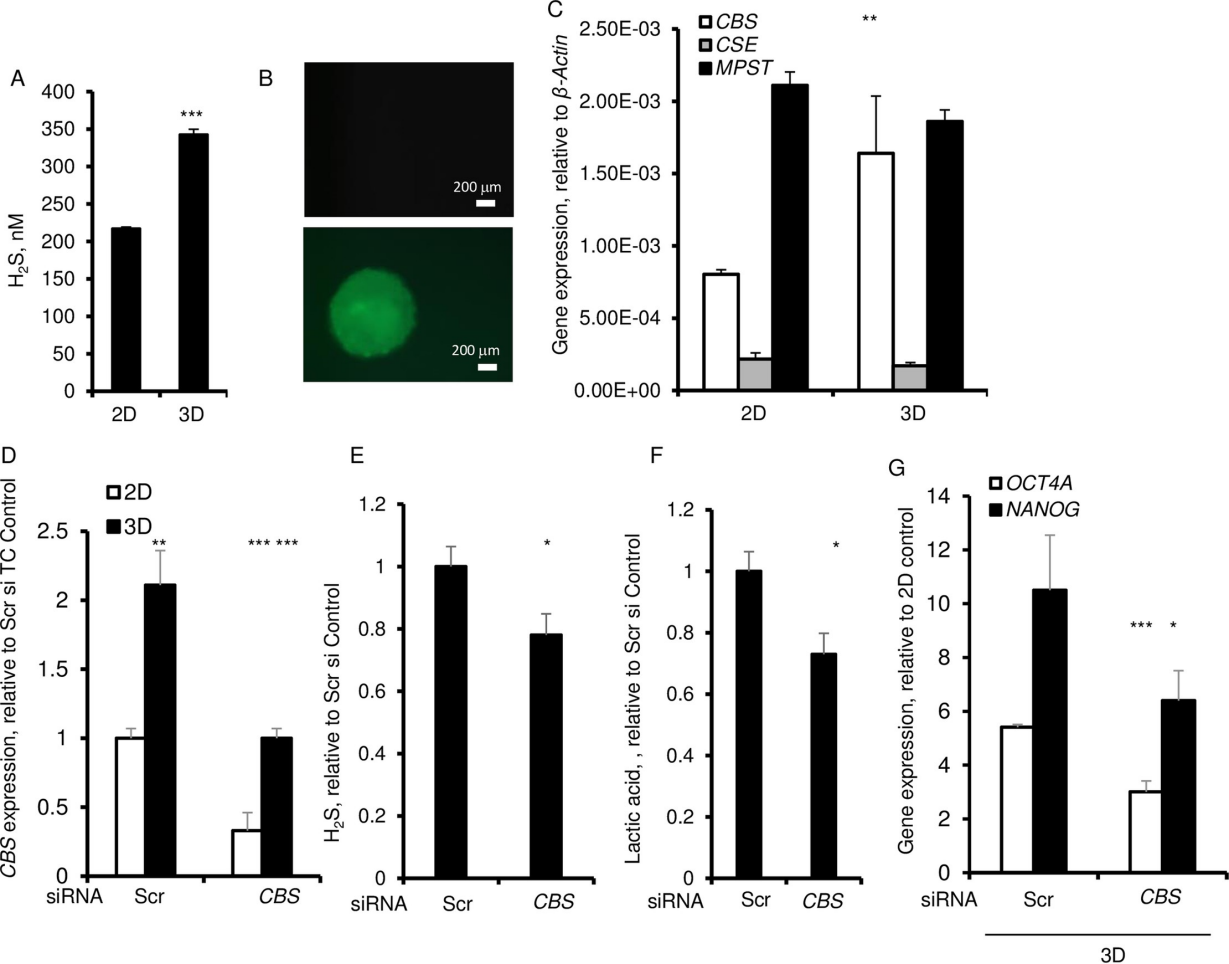
*CBS* dependent regulation of H_2_S and expression of pluripotency gene, *OCT4* in 3D spheroids. A. H_2_S released by HDF after 6 day of culture. B. H_2_S stained with 5 μM H_2_S fluorescent probe, HSN2. C. qPCR of expression of H_2_S-synthesizing enzymes, *CBS*, *CSE*, *MPST*. *β-ACTIN* was used as a housekeeping control. Cells were transfected with *CBS* siRNA or scrambled (Scr) control siRNA. D. qPCR of expression of *CBS*. E. Amount of H_2_S in culture media. F. Amount of lactic acid in culture media. G. qPCR of expression of *OCT4* and *NANOG* after 72 h of culture. *β-ACTIN* was used as a housekeeping control. Data are presented as the mean ± SD from 3–6 independent experiments. Statistical significance is shown as * (p≤0.05), ** (p ≤0.005), and *** (p ≤0.0005) in 3D as compared to 2D cultured cells.

H_2_S increases *TET1* expression while it represses DNA methyltransferases [26]. Given that in 3D cultures the *TET1* was increased and the *DNMT1* was suppressed, we then examined the level of H_2_S that was released into the culture supernatants. H_2_S was significantly higher in 3D culture supernatants and within spheroids as evidenced by staining with the fluorescent probe, HSN2 (Fig 3A and 3B). There was no stainable H_2_S within the monolayer cultures (Fig 3B). Biosynthesis of H_2_S is mainly regulated by three enzymes, namely, cystathionine β synthase (CBS), cystathionine-γ-lyase (CSE), and 3-mercaptopyruvate sulfurtransferase (MPST). No significant difference was found in the level of either *CSE* or *MPST* mRNA in cells cultured in 3D as compared with those cultured under 2D conditions (Fig 3C). However, there was a significant increase in the *CBS* mRNA in 3D cultured cells supporting the idea that H_2_S production in spheroids is mainly regulated by *CBS* in 3D cultures (Fig 3C). To validate this idea, we silenced *CBS* by its siRNA by ~ 60% in monolayer and 3D cultures (Fig 3D). The *CBS* silencing significantly decreased H_2_S (Fig 3E). Moreover, this silencing further showed that H_2_S was essential to the lactic acid production and to the expression of *OCT4* and *NANOG* (Fig 3F and 3G).

**Fig 4:**
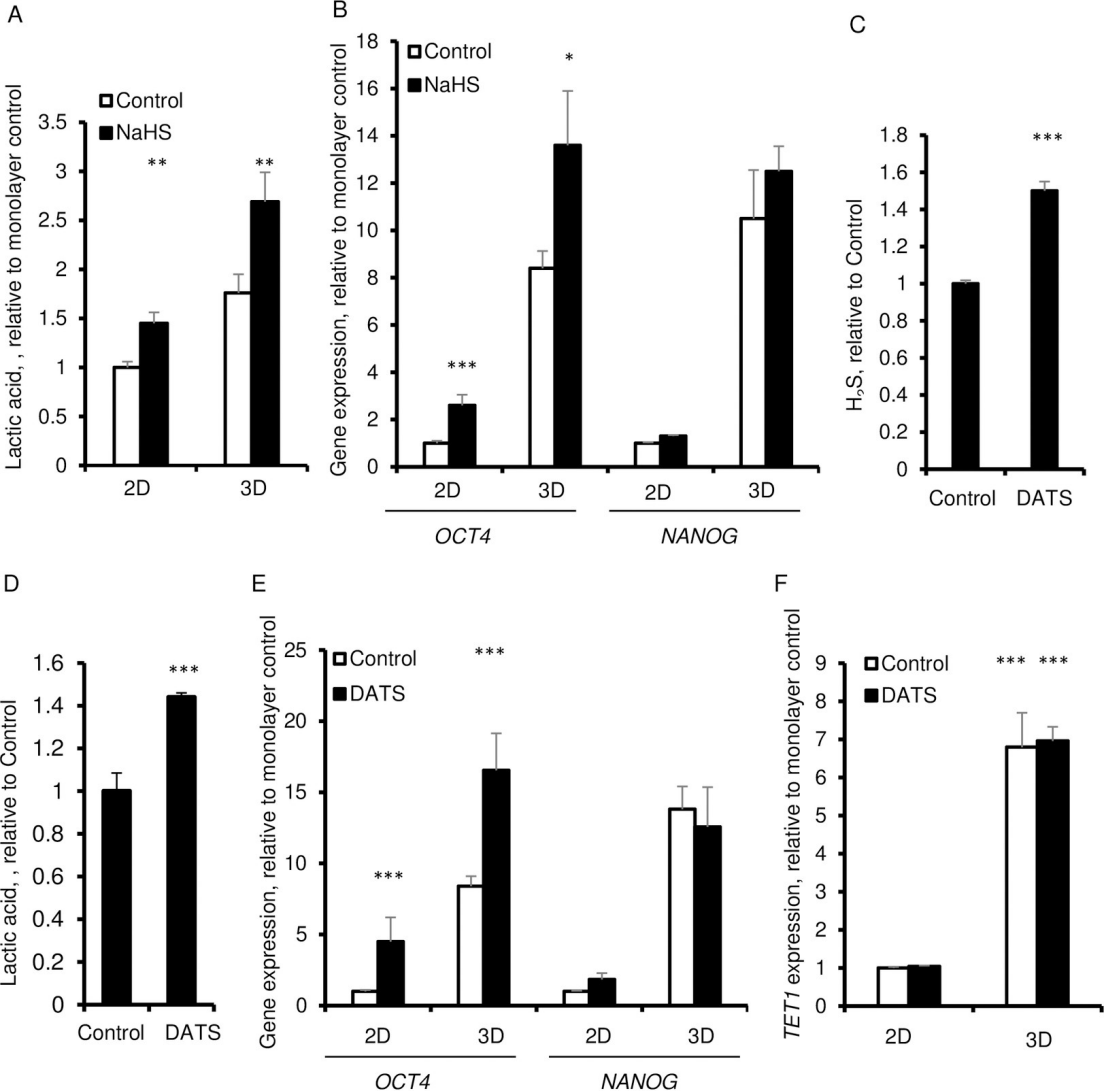
Effect of H_2_S donors, NaHS and DATS on lactic acid production and expression of *OCT4* and *NANOG* in 3D spheroids. A. Extracellular lactic acid in presence of 50 μM NaHS after 6 days. B. qPCR of *OCT4* and *NANOG* in presence of 100 μM NaHS for 6 days. C-G. HDFs were cultured for 6 days in in 2D and 3D in the absence and the presence of 50 μM DATS. C. Extracellular H_2_S in presence of 50 μM DATS after 6 days. D. Extracellular lactic acid in presence of 50 μM DATS after 6 days. E. qPCR of *OCT4* and *NANOG*. *β-ACTIN* was used as a housekeeping control. F. qPCR of *TET1*. Data are presented as the mean ± SD from 3–6 independent experiments. *β-ACTIN* was used as a housekeeping control. Statistical significance is shown as * (p≤0.05), ** (p ≤0.005), and *** (p ≤0.0005) in 3D as compared to 2D cultured cells.

To further establish the importance of H_2_S in causing glycolysis, fibroblast cells were treated with 100 μM of the H_2_S donor, NaHS. This treatment led to increased secretion of lactic acid not only in 3D cultures but in 2D cultures as well (Fig 4A). More importantly, cells in 3D cultures in the presence of 100 μM NaHS showed increased expression of *OCT4* which indicates that H_2_S is essential for reprogramming under such culture conditions (Fig 4B). However, the 3D induced increase in NANOG was not further elevated upon addition of NaHS (Fig 4B). In 2D cultures the response was also limited to a significant increase in *OCT4* without an increased in *NANOG* expression (Fig 4B). To further validate these findings, we used a different H_2_S donor, garlic-derived diallyl trisulfide (DATS). Unlike NaHS, DATS is stable with minimal spontaneous H_2_S release. The slow release of H_2_S occurs in the presence of thiols [27, 28]. DATS significantly elevated hydrogen sulfide level in the culture supernatant as compared to the control cells that did not receive DATS (Fig 4C). Similar to NaHS, DATS increased the production of lactic acid and enhanced the expression of *OCT4*, without any effect on the expression of *NANOG* or *TET1* in 2D or 3D cultures (Fig 4D–4F).

### H_2_S increases the osteogenic potential of HDFs grown in 3D cultures

**Fig 5:**
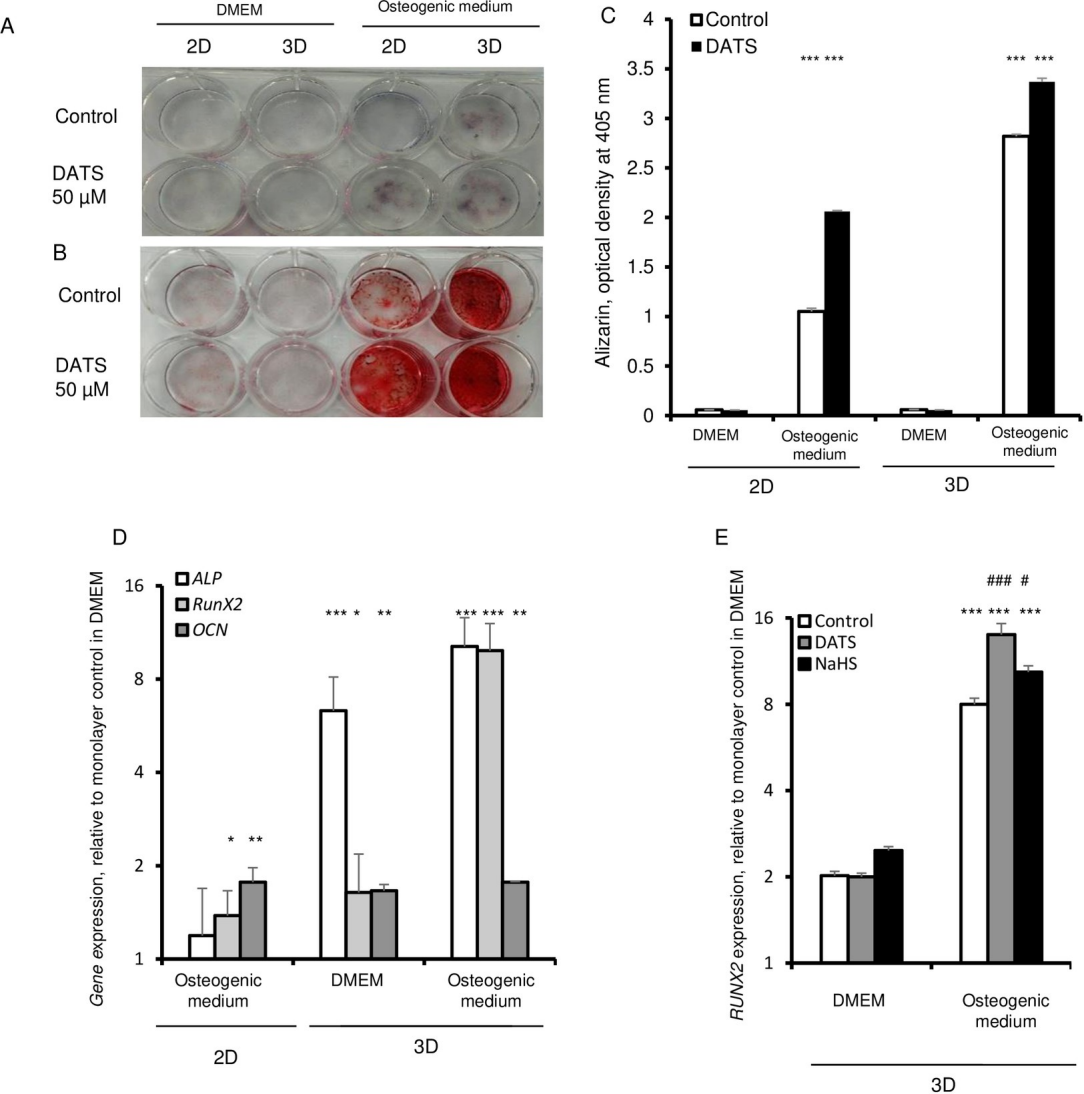
H_2_S promotes osteogenic differentiation in 3D spheroids. HDFs were cultured in 3D in the absence and presence of 50 μM DATS for 6 days. The cells were then cultured in DMEM and osteogenic conditions for up to 21 days. A. Alizarin Red staining. B. Alkaline phosphatase staining. C. Densitometric analysis of Alizarin staining. D. mRNA expression levels of osteogenic markers such as alkaline phosphatase (*ALP*), osteocalcin (*OCN*) also referred to as bone γ-carboxyglutamic acid-containing protein or BGLAP, and *RUNX2*, and expression of were evaluated in 2D and 3D cultures maintained in DMEM and culture condition allowing osteogenic differentiation. Expression levels were normalized to the expression level in monolayer (2D) cells cultured in DMEM. E. HDFs were cultured in 3D in the absence and presence of 50 μM DATS or 100 μM of NaHS for 6 days and then subjected to osteogenic differentiation. qPCR analysis of *RUNX2* gene expression normalized to the expression level in 2D cultured cells. Statistical significance is shown as * (p≤0.05), ** (p ≤0.005), and *** (p ≤0.0005) in 3D as compared to 2D cultured cells.

Similar to mesenchymal stem cells, fibroblasts have the potential to generate mesodermal lineages *in vitro* after culture with differentiation inducing agents [29]. Therefore, we examined the differentiation capacity of fibroblasts that were cultured as 3D spheroids. HDFs were cultured as 2D monolayers and 3D spheroids in presence of αMEM medium, containing knockout serum. After 6 day of culture, the medium was changed to an osteogenic trans-differentiation medium with the addition of high-glucose, 15% FBS, 10^-7^M dexamethasone, 10 mM glycerophosphate, and 0.05 mM ascorbic acid [30, 31]. Alkaline phosphatase (ALP) is a generally used as a marker for early osteogenic differentiation, whereas mineralization is a characteristic of late osteogenic differentiation. Thus, we assessed the osteogenic differentiation on day 30 of culture by analyzing the alkaline phosphatase (ALP) activity, and assessed mineralization by Alizarin Red S staining. Under osteogenic conditions, monolayer cells exhibited very low ALP and Alizarin Red S staining (Fig 5A and 5B). However, ALP positive staining was readily visible in 3D culture and, particularly, this staining was more intense in 3D treated with DATS (Fig 5A–5C). Similar to the ALP activity, Alizarin Red S staining was weak under non-osteogenic conditions. The qualitative and quantitative Alizarin Red staining analysis of mineralization showed intense mineralization in 3D spheroid cells under osteogenic conditions (Fig 5B and 5C). The different osteogenic target genes were analyzed, including *RUNX2*, *ALP*, and *OCN* (Fig 5D). The results indicated significant expression of *ALP* and *RUNX2* in 3D compared to 2D cultured in the osteogenic inducing media. The expression level of *OCN* did not differ between 2D and 3D under osteogenic condition. Mineralization was most significantly increased, as evidenced by Alizarin Red S staining, in 3D cultures treated with DATS in both non-osteogenic and osteogenic media (Fig 5C). Furthermore, DATS significantly increased the expression of *RUNX2*, which is a key transcription factor associated with osteoblast differentiation (Fig 5E). Cells maintained under non-osteogenic condition failed to show robust osteogenic differentiation (Fig 5A–5E). Treatment of 3D cultures with NaHS led to a significant increase in the expression of *RUNX2* in non-osteogenic and osteogenic conditions (Fig 5E). Our results support the idea that H_2_S is essential to the *RUNX2* mediated osteogenic potential of fibroblasts.

## Discussion

Cell aggregates are often used as an important tool in the studies of tissue regeneration due to re-establishment of cell-cell contacts, cell functions, and the enhanced survival in *in vitro* conditions [32]. The most common methods for scaffold-free spheroid formation are hanging-drop method, gentle rotational stirring, and magnetic levitation of suspensions of dispersed cells [33–36]. Spheroids can also be produced by preventing cells from attaching to the culture substrate. Takezawa and co-authors demonstrated that by day 2 confluent normal adult human dermal fibroblasts detached from the thermoresponsive non-adhesive substrates, which is comprised of poly-N-isopropyl acrylamide (PNIPAAm) and type I collagen, and form floating cell sheets that develop into multi-cellular spheroid colonies [37]. The spheroids had cuboidal cells at the center which were covered with squamous cells adherent to each other by gap and tight junctions. Consistent with prior reports, our results reveal that dermal fibroblasts that were forced to develop spheroids in low adherent culture conditions were only partially reprogrammed and expressed *OCT4*, *SOX2*, and *NANOG* and not the *KLF4* and *c-MYC* [16].

*OCT4* is essential for the generation of induced pluripotent state and is crucial for early phase of reprogramming [38]. Here, we present that the expression of *OCT4* is regulated in 3D cultures by gaseous signaling molecule, H_2_S. H_2_S is generated by cystathionine β-synthase (CBS), cystathionine γ-lyase (CSE), and 3-mercaptopyruvate sulfurtransferase (MPST). Amongst these enzymes, only the expression of *CBS* was increased in 3D spheroids. The expressions of *CSE* and *MPST* remained unaltered. The siRNA-mediated silencing of *CBS* in spheroid cultures of adult human fibroblasts diminished the expression of *OCT4*. In the presence of H_2_S donors, NaHS and DATS, aggregation of fibroblasts in 3D cultures significantly enhanced the expression of *OCT4* and did not alter the expression of *NANOG*. The findings that expression of *OCT4* is significantly upregulated by H_2_S donors and downregulated by *CBS* silencing highlight the importance of H_2_S in the early phase of the reprogramming process. This reprogramming appears to be controlled by PI3K/Akt signaling, which is a crucial early stage in human fibroblast reprogramming that leads to the phosphorylation of the OCT4 at threonine 235 [39, 40]. OCT4 phosphorylation increases its stability and facilitates its nuclear re-localization and interaction with SOX2, which promotes the transcription of the core stemness genes [41]. Our data revealed that 3D culture conditions modify the expression of *TET1* and *DNMT1* that participate in methylation of genes which are essential to the reprogramming [23]. The 3D culture environment significantly increased *TET1* and significantly decreased *DNMT1*. Our findings are consistent with such changes in the expression of enzymes that are involved in DNA methylation during reprogramming. It is recently shown that erasure of DNA methylation at distal and proximal elements of *OCT4* and *NANOG* regulatory region promotes *OCT4* and *NANOG* overexpression and disruption of expression of *DNMT*s increases cellular plasticity [25, 42, 43].

Consistent with the proteomic studies which show the need for metabolic reprogramming to aerobic glycolysis in 3D cultures, we also show that 3D cultures promote the expression of *PDK4* and, *PC* as well as *LDH*, and glycolytic export of lactic acid, which is a required step towards reprogramming and pluripotency [13, 44]. These findings are in conjunction with earlier results that show that overexpression of *CBS* induces a change in the energetic metabolism, and more specifically drives the glycolysis and pyruvate metabolism [45]. Moreover, the continuous release of H_2_S has been shown to increase glycolysis in diverse types of cells [46, 47].

Spheroid culture method was shown to improve differentiation potential of MSCs compared to monolayer culture [48]. Thus, 3D culture condition maintained hDPSCs ability to differentiate toward osteogenic lineage [12]. Ours results also indicate that 3D culture conditions favor osteogenic differentiation of human dermal fibroblasts. H_2_S regulates self-renewal of mesenchymal stem cells and enhances the effectiveness of cell therapy for tissue generation [49–51]. H_2_S also drives the osteogenic differentiation of bone marrow mesenchymal stem cells and RANKL-induced osteoclastogenesis [50, 52]. Our findings support the idea that 3D culture enhances the osteogenic potential of fibroblasts through mechanisms that require *CBS* overexpression and production of H_2_S. The increase in osteogenic potential appears to be dependent on hydrogen sulfide since differentiation potential increases in the presence of the H_2_S donor, DATS. H_2_S is known to increase nuclear accumulation and transactivation of RUNX2, a master transcription factor that is essential to osteogenesis [53]. The H_2_S donor, NaHS, is known to increase the sulfhydration of cysteine residues in the DNA binding domain of RUNX2 [53, 54]. Given that the expression of *CBS* increases during osteoclast differentiation, it appears that H_2_S is a required factor in osteogenesis, and the use of H_2_S donors represents a promising direction for biomedical applications of 3D cultured human fibroblasts in tissue repair.
